# Post-Exertional Malaise in Post-COVID-19 Syndrome: A Shift in the Frequency Across Pandemic Phases

**DOI:** 10.3390/jcm15082948

**Published:** 2026-04-13

**Authors:** Alaa Ghali, Christian Lavigne, Maria Ghali, Valentin Lacombe

**Affiliations:** 1 Outpatient Department, Doue-En-Anjou Hospital, F-49700 Doue-En-Anjou, France; 2 Department of Internal Medicine and Clinical Immunology, Angers University Hospital, F-49100 Angers, France; chlavigne@chu-angers.fr; 3 Department of General Medicine, University of Angers, F-49000 Angers, France; dr.maria.ghali@gmail.com; 4 POPS, SFR ICAT, University of Angers, F-49000 Angers, France; 5 Department of Internal and Polyvalent Medicine, Haut-Anjou Hospital, F-53200 Chateau-Gontier, France; lacombe.valentin31@gmail.com

**Keywords:** post-COVID-19 syndrome, myalgic encephalomyelitis/chronic fatigue syndrome, post-exertional malaise, pre-omicron phase, vaccination

## Abstract

**Background:** Post-exertional malaise (PEM), which is the cardinal feature of myalgic encephalomyelitis/chronic fatigue syndrome (ME/CFS), is also reported in a proportion of patients with post-COVID-19 syndrome (PCS). Our objective was to identify determinants that may be linked to the emergence of PEM in PCS patients. **Methods:** Patients fulfilling the World Health Organization definition for PCS who attended the post-COVID unit of the Internal Medicine Department of Angers University Hospital, France, between June 2020 and December 2023 were included retrospectively. Their medical records were reviewed to extract information on COVID-19 infection history, characteristics of post-exertional malaise (PEM), fatigue severity, and relevant epidemiological variables. **Results:** The study included 220 patients, grouped according to whether post-exertional malaise was present (PCS/PEM+) or absent (PCS/PEM–). PEM was observed in 26.4% of patients and was significantly linked to earlier COVID onset in 2020/2021 (OR 5.68 (95% CI: 1.66–19.45), *p* = 0.006), as well as higher fatigue levels (OR 2.07 (95% CI: 1.22–3.50), *p* = 0.007). **Conclusions:** Patients who contracted COVID-19 during the pre-Omicron period reported PEM more frequently than those infected in later waves. This observation could reflect differences in viral characteristics following the emergence of the Omicron variant; however, alternative explanations—such as increasing vaccination coverage, accumulating post-infectious immunity, or other unmeasured factors—cannot be ruled out. Based on the observed link between PEM and symptom severity, PCS patients should be systematically assessed for the presence of PEM.

## 1. Introduction

According to the World Health Organization (WHO), post-COVID-19 syndrome (PCS), also referred to as long COVID, is defined as the persistence or onset of new symptoms occurring three months after the initial SARS-CoV-2 infection and lasting for at least two months, without an alternative diagnosis [1]. PCS is estimated to affect 10–20% of non-hospitalized patients [2], and its prevalence among vaccinated individuals is reported to range between 10 and 12% [3].

PCS involves a wide variety of symptoms, many of which resemble those described in myalgic encephalomyelitis/chronic fatigue syndrome (ME/CFS), in particular severe fatigue, neurocognitive impairment, sleep disturbances, orthostatic intolerance, and post-exertional malaise (PEM) [4]. PEM, a cardinal feature of ME/CFS, corresponds to post-exertional exhaustion after physical, cognitive, emotional, or orthostatic exertion that would previously have been tolerated. PEM is accompanied by a worsening of some or all baseline symptoms [5,6] and is reported to be associated with disability [7] and poorer outcomes for patients [8].

PCS and ME/CFS share several other features. Both occur after viral infections, most notably Epstein–Barr virus in the case of ME/CFS [9], primarily affect healthy, active women [10], and are characterized by a marked worsening of symptoms following minimal exertion [4]. They also share similar associated comorbidities such as mast cell activation syndrome (MCAS) [11,12], mast cell activation disorder (MCAD) [13], and postural orthostatic tachycardia syndrome (POTS) [14,15]. Furthermore, some pathophysiological mechanisms are common in both PCS and ME/CFS patients, including inappropriate immune response, systemic and neuro-inflammation, and mitochondrial dysfunction [16]. The lack of a disease-specific biomarker [17], the absence of any curative therapy, and the central role of pacing in symptom management [18] further illustrate the similarities between PCS and ME/CFS. Owing to the overlapping between the two conditions, some authors have suggested adopting the term ‘post-COVID-19 ME/CFS’ [19].

PEM is reported in only a subset of PCS patients, emphasizing the heterogeneity of PCS and suggesting that PEM-positive patients may represent a distinct clinical phenotype. Given the adverse impact of PEM on the quality of life of ME/CFS patients, our objective was to identify determinants that may be linked to the emergence of PEM in PCS patients.

## 2. Patients and Methods

### 2.1. Ethics

Ethical approval was obtained from the Ethics Committee of Angers University Hospital on 9 July 2024 (2024/154), and the study was carried out in accordance with the Declaration of Helsinki. Authorization for data collection was granted by the French Data Protection Authority.

### 2.2. Study Population

Patients fulfilling the WHO definition for PCS [1] who attended the post-COVID unit of the Internal Medicine Department of Angers University Hospital, France, in the period between June 2020 and December 2023 were retrospectively included in the study. Data were extracted from 253 medical records.

### 2.3. Exclusion Criteria

We excluded medical records of patients with a pre-existing diagnosis of ME/CFS (*n* = 3) or reporting PEM prior to COVID infection due to conditions other than ME/CFS, such as multiple sclerosis (*n* = 2). Records with a doubtful diagnosis of associated comorbidities—particularly POTS and MCAS/MCAD—were also excluded (*n* = 4), as were those with insufficient documentation of PEM assessment (*n* = 5), absence of an OH assessment (*n* = 3), or errors in completing the FSS questionnaire (*n* = 6). We thus retained 220 medical records.

### 2.4. Study Design

All included patients were assessed by a single physician, who also established the diagnosis, and were systematically screened for PEM, which was identified on the basis of the Canadian Consensus Criteria (CCC) [5] and the International Consensus Criteria (ICC) [6] for ME. Fatigue severity was also evaluated for all patients.

Patients’ medical records were reviewed to extract relevant epidemiological variables and information on COVID-19 infection history, especially the date of onset, vaccination status, and related clinical manifestations. When present, information concerning PEM was documented. Data on associated comorbidities, including POTS, MCAS/MCAD, and fibromyalgia, were collected.

### 2.5. PEM Definition

PEM corresponds to a pathological post-exertional response, not a simple increase in fatigue level. It involves the occurrence of post-exertional exhaustion after exposure to physical, cognitive, emotional, or orthostatic stressors that were previously well tolerated. This post-exertional exhaustion is accompanied by a worsening of baseline symptoms, including cognitive dysfunction, autonomic manifestations, neurosensory intolerance, and sleep disturbance, as well as the possible emergence of new or unusual symptoms. Its onset can be immediate or delayed by several hours or longer after the stressor. The duration of PEM varies widely between patients and within the same patient, lasting from several days to weeks or even months. A duration of at least 24 h is required for PEM to meet diagnostic criteria [5,6,20].

### 2.6. PEM Analysis

Data on PEM characteristics were collected using a standardized questionnaire that we designed and previously applied in ME/CFS patients [21].

### 2.7. Measures

#### 2.7.1. Fatigue Assessment

Fatigue severity was assessed using the Fatigue Severity Scale (FSS) [22], a validated nine-item instrument measuring the functional impact of fatigue on daily activities. The FSS score is calculated as the mean of the nine items. FSS scores ≥ 4 indicate clinically significant fatigue [23].

#### 2.7.2. POTS Assessment

POTS was evaluated by means of the active standing test and/or the head-up tilt table test. The active standing test involves measuring blood pressure and heart rate after at least 5 min in the supine position, followed by repeated measurements at 1, 3, 5, and 10 min of standing. POTS is characterized by a heart rate increase on standing ≥30 bpm without accompanying orthostatic hypotension. During the passive head-up tilt table test, blood pressure and heart rate were assessed in the supine position and again after tilting the patient to an angle exceeding 60° [24].

#### 2.7.3. OH Assessment

The assessment of OH was realized by measuring the blood pressure and heart rate after 5 min in the supine position and 3 min after moving to a standing position. A decrease in blood pressure ≥ 20 mm Hg systolic or ≥10 mm Hg diastolic within 3 min of standing from the supine position is diagnostic of OH [25].

### 2.8. Patients’ Grouping

Included patients were divided into two groups for comparative analysis according to the presence (PCS/PEM+) or absence (PCS/PEM−) of PEM.

### 2.9. Statistical Analysis

Quantitative variables (expressed as medians and interquartile ranges) were compared using Student’s *t*-test. Categorical variables (reported as counts and percentages) were compared using the Chi-square test. Multivariable analysis was performed using binary logistic regression, adjusting for age, sex, and variables significantly associated with PEM in univariate analysis. Odds ratios (ORs) were reported with their 95% confidence intervals (95% CI). A two-sided *p*-value < 0.05 was considered statistically significant. Statistical analyses were conducted using GraphPad Prism version 6.01 and Jamovi version 2.3.9.

## 3. Results

A total of 220 patients meeting the WHO definition of PCS [1] were included in this retrospective study.

The majority were women (176/220, 80.0%), and the median age at COVID-19 infection was 42 (34–50) years. The median time from infection to PCS diagnosis was 15 (9–23) months.

Most infections occurred during the years 2020–2021 (140/220, 63.6%), and a large number of patients (144/220, 65.0%) were not vaccinated. PEM was identified in 58/220 (26.4%) patients. Fatigue was reported by 216/220 (98.1%) patients, with a median FSS score of 6.3 (5.4–7.0). Other frequent symptoms were myalgia (63.6%), headache (56.3%), cognitive deficits (54.0%), and dyspnea (52.7%) (Table 1).

**Figure 1:**
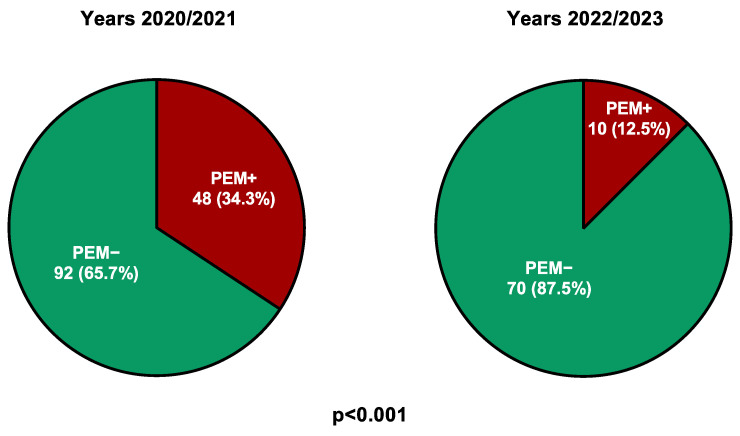
Frequency of post-exertional malaise in post-COVID syndrome patients according to the COVID infection period. PCS/PEM+: group of patients with PEM; PCS/PEM−: group of patients without PEM.

The comparison of patients in the univariate analysis, according to the presence (PCS/PEM+) or absence (PCS/PEM−) of PEM, revealed a significant association between the occurrence of PEM and the onset of infection during the years 2020/2021 (*p* < 0.001, Figure 1), the absence of vaccination (*p* = 0.05), higher FSS scores (*p* < 0.001), more frequent occurrence of POTS (*p* < 0.001), palpitations (*p* < 0.001), and orthostatic hypotension (*p* < 0.001) (Table 1).

In multivariate logistic regression analysis, two factors remained significantly associated with PEM in PCS patients: COVID-19 infection occurring during the years 2020–2021 (OR 5.68 (95% CI: 1.66–19.45), *p* = 0.006), and higher FSS scores (OR 2.07 (95% CI: 1.22–3.50), *p* = 0.007, Table 2).

## 4. Discussion

The study population consisted of 220 patients having PCS, the majority of whom were female with a median age at COVID infection of 42 (34–50) years. The predominance of female patients (80%) was consistent with recently reported data [26,27,28]. Almost all patients (98.1%) complained of fatigue as observed in previous works [27,28]. The high mean FSS score of 6.3 (5.4–7.0) indicated severe fatigue.

The observed PEM prevalence in the current study (26.4%) closely aligned with that reported by a recent study (24%) [29] and fell within the wide range reported in the literature (17.5%–94.8%) [30,31,32,33,34,35], which may reflect differences in study design and timing (pre-Omicron vs. Omicron periods), patient selection criteria (inpatient vs. outpatient setting, vaccination status, underlying comorbidities), and the criteria used to diagnose PEM. In our study, the CCC and ICC for ME diagnosis were used to identify PEM in all PCS patients [5,6].

POTS and POTS-like symptoms such as palpitations and tachycardia are frequent in both PCS and ME/CFS patients [4,36,37]. In our study population, the prevalence of POTS at 7.3% is in accordance with that observed by Ormiston et al. [38], which found that 2–14% of PCS patients develop POTS within 6–8 months of the acute infection. In a large prospective cohort of highly symptomatic long COVID patients, 31% were diagnosed with POTS using standardized autonomic testing [39]. The prevalence of OH (10.3%) in our patients was consistent with the results of other studies, which reported OH prevalence in PCS ranging from 8 to 13.8% [40,41,42]. Similarly, in our study, palpitations were observed in 26.3% of patients, which in line with the ranges of 25 to 50% reported by Ståhlberg et al.; however, a recent meta-analysis of nearly 3 million patients found that 18% of SPC patients reported palpitations [43]. In the univariate analysis, POTS and OH were more frequently observed in patients with PEM; however, these associations did not persist in multivariate analysis. This pattern may reflect the influence of shared underlying factors rather than an independent relationship between these conditions. In particular, the period of infection could act as a common determinant, as the later phase of the pandemic—largely corresponding to the Omicron period—was associated with a lower prevalence of PEM in our cohort and may have also been characterized by fewer dysautonomic manifestations.

Our analysis showed a clear temporal difference between the periods 2020/2021 and 2022/2023, during which the most notable epidemiological shift was the emergence and subsequent predominance of the Omicron variant. In France, the dominant SARS-CoV-2 strain was the wild-type throughout the year 2020, followed by the Alpha variant from January to May 2021, and then the Delta variant from June to December 2021. From 2022 onward, the Omicron variant has persisted until today [44]. Considering that the wild-type, Alpha, and Delta variants have been reported to yield similar long term COVID-19 sequelae [45], and given the absence of genomic identification of SARS-CoV-2 variants in our study, we compared PCS patients with and without PEM who contracted COVID infection before the emergence of the Omicron variant (2020/2021) with those infected during the omicron period (2022/2023).

In our study, PEM was six times more frequent among PCS patients infected in the pre-Omicron period relative to those infected in the Omicron period. Moreover, the presence of PEM was associated with higher FSS scores, indicating more severe fatigue in these patients, as previously reported [4,46,47]. Thus, non-Omicron breakthrough infections were associated with a higher frequency of PEM and, consequently, with more severe forms of PCS than Omicron infections. This finding is in line with results of multiple cohort studies and meta-analyses showing that the risk of persistent symptoms—especially fatigue and PEM—is highest following infection with pre-Omicron variants and lower after Omicron infection [48,49,50,51].

One may wonder whether the lower proportion of patients reporting PEM in the later phase of the pandemic could in part be related to shifts in circulating viral genotypes. Although no studies, to our knowledge, have specifically examined the relationship between SARS-CoV-2 genotype or variant and the occurrence of PEM as an isolated symptom, several works have reported that the pre-Omicron period was associated with more prolonged and severe fatigue, a symptom closely related to PEM [52,53,54,55].

Nevertheless, other factors that evolved over the course of the pandemic may also have contributed to the temporal differences observed between the 2020/2021 and 2022/2023 periods. Greenhalgh et al. concluded that the apparent variant differences are heavily confounded by vaccination status, prior immunity, and changes in acute COVID-19 management over time [56]. Wong et al. [57] reported that the Omicron variant had similar intrinsic severity to the wild-type strain and attributed the reduced effective severity of COVID-19 in Omicron cases to vaccination rather than to the viral strain itself. A multicenter prospective study found that the pre-Delta cohort exhibited more prolonged severe fatigue (16.7% vs. 11.5% vs. 12.3% for pre-Delta, Delta, and Omicron); however, after adjusting for vaccination status, these differences were no longer significant, suggesting vaccination rather than variant-specific pathophysiology may explain the observed differences [53]. In the RECOVER study [55], subtype 5—the most severe phenotype characterized by the worst quality of life, physical health, and daily function—was more prevalent among individuals infected before the Omicron variant or before vaccination, potentially suggesting distinct mechanisms of disease.

In our study, PEM occurrence in PCS patients was associated with a higher proportion of unvaccinated individuals (*p* = 0.05); however, this association did not persist in multivariate analysis. This lack of statistical significance may partly reflect limited statistical power, as vaccinated individuals represented a minority of patients in our sample. The available evidence on post-exertional malaise (PEM) and vaccination status in post-COVID patients is limited, with most studies focusing on overall SPC symptoms rather than PEM specifically. However, vaccination appears to have differential effects on post-COVID syndrome phenotypes. Two recent meta-analyses found that receiving two-dose vaccine was associated with a reduced risk of persistent fatigue and other post-COVID symptoms compared with no vaccination [58,59]. In addition, a recent cohort study demonstrated that individuals vaccinated and subsequently infected with Omicron had a substantially lower risk of post-COVID symptoms—including PEM—than those infected with earlier variants (wild-type, Alpha, or Delta), even after adjustment for vaccination status and number of doses [60]. In contrast, a recent multicenter cohort study found that vaccination was associated with reduced risk of the Respiratory and the Cognitive Clusters, but not the Fatigue Cluster [61]. Overall, as shown in a large study including 441,583 veterans [62], viral changes together with the role of vaccination could have contributed to the shift in PEM occurrence between the pre- and post-Omicron periods. Our study did not demonstrate an independent association between vaccination and PEM. The possible role of vaccination is requiring confirmation in dedicated studies.

Beyond epidemiological considerations, differences in viral biology between SARS-CoV-2 variants may also contribute to the variation in PEM prevalence observed across pandemic phases. Experimental studies suggest that the Omicron variant exhibits reduced neurotropism and neurovirulence compared with earlier variants such as the ancestral strain or Delta, resulting in lower levels of neuro-inflammatory activation [63,64]. In addition, the Omicron spike receptor-binding domain (RBD) appears to exhibit lower pathogenicity but higher antigenicity than earlier variants, for which pathogenicity decreases in the order: wild-type > Gamma > Delta > Omicron [65]. Its higher antigenicity promotes a more effective antiviral immune response. Moreover, Omicron is also associated with reduced clinical severity due to its low pro-inflammatory and IL-6-stimulating capacity, together with increased induction of IFN-γ and IL-4 compared with previous variants [65]. Since IL-6 and other pro-inflammatory cytokines contribute to mitochondrial dysfunction, endothelial injury, and skeletal muscle damage—key mechanisms implicated in PEM pathophysiology—the reduced inflammatory profile of Omicron may lessen these subsequent effects and thereby reduce the likelihood of PEM. Furthermore, despite partial escape from neutralizing antibodies, cellular antiviral immunity against Omicron remains largely preserved, enabling more efficient viral control and possibly reducing the persistence of immune dysregulation following infection [66]. Taken together, these differences in viral biology may contribute to a lower propensity to trigger the immune and metabolic disturbances underlying PEM. Nevertheless, the reduced pathogenicity of Omicron is confounded by the higher level of population immunity (from vaccination and prior infection) during the Omicron period [62]. Given that, to our knowledge, no studies have directly examined variant-specific effects on mitochondrial function, skeletal muscle bioenergetics, or exercise physiology—the proximate mechanisms underlying PEM—and considering the experimental nature of some studies investigating virus-specific mechanisms, these interpretations should be regarded as hypothesis-generating and require confirmation in dedicated studies.

This study was limited by the lack of genomic data on SARS-CoV-2 variants. A further limitation was its retrospective design; however, all patients were evaluated and diagnosed by the same physician and underwent an identical standardized assessment, including evaluation of PEM, fatigue, and relevant comorbidities. Despite these limitations, the study benefits from a substantial sample size and from the consistent identification of PEM in all patients using uniform criteria [5,6].

## 5. Conclusions

PEM was more common in PCS patients infected prior to the emergence of Omicron than in those infected during the Omicron phase. This likely reflects viral biology changes associated with Omicron. Although we did not observe an independent association between vaccination and PEM, a potential contribution of vaccination cannot be excluded and warrants further investigation. In addition, our study confirms that PEM occurrence in PCS patients is linked to more severe fatigue and adversely affects patients’ health status and quality of life. Therefore, it is essential to include PEM in the standard clinical evaluation of patients with PCS. Early identification will facilitate the early implementation of pacing strategies to minimize PEM-triggering factors and improve the quality of life of individuals living with PCS.

## Figures and Tables

**Table 1:** Comparison of features of post-COVID syndrome patients according to the presence or absence of post-exertional malaise.

	Whole Population (*n* = 220)	PCS/PEM+ (*n* = 58)	PCS/PEM− (*n* = 162)	*p*-Value
General features				
Gender (female)	176 (80.0%)	46 (79.3%)	130 (80.2%)	0.88
Age at COVID-19 onset (years)	42 (34–50)	43 (34–48)	41 (33–51)	0.53
Time from COVID-19 onset to PCS diagnosis (months)	15 (9–23)	15 (10–23)	15 (8–22)	0.27
Period of COVID-19 infection				<0.001
2020/2021 (*n*, %)	140 (63.6%)	48 (82.8%)	92 (56.8%)	
2022/2023 (*n*, %)	80 (36.4%)	10 (17.2%)	70 (43.2%)	
Vaccinated patients (*n*, %)	76 (34.5%)	14 (24.1%)	62 (38.2%)	0.05
Associated comorbidities (*n*, %)				
POTS	16 (7.3%)	10 (17.2%)	6 (3.7%)	<0.001
MCAS/MCAD	138 (62.7%)	37 (63.7%)	101 (62.3%)	0.84
Fibromyalgia	11 (5.0%)	3 (5.2%)	8 (4.9%)	0.94
Symptoms (*n*, %)				
Fatigue	216 (98.1%)	57 (98.2%)	159 (98.1%)	0.95
FSS score	6.3 (5.4–7.0)	6.8 (6.2–7.0)	6.2 (5.2–6.8)	<0.001
Palpitations	70 (31.8%)	31 (53.4%)	39 (24.0%)	<0.001
Orthostatic hypotension	29 (13.1%)	16 (27.5%)	13 (8.2%)	<0.001
Chest pain	33 (15.0%)	9 (15.5%)	24 (14.8%)	0.90
Dyspnea	116 (52.7%)	31 (53.4%)	85 (52.4%)	0.90
Cough	54 (24.5%)	12 (20.6%)	42 (25.9%)	0.43
Cognitive deficits	119 (54.0%)	36 (62.0%)	83 (51.2%)	0.16
Headache	124 (56.3%)	36 (62.0%)	88 (54.3%)	0.30
Vertigo	96 (43.6%)	30 (51.7%)	66 (40.7%)	0.15
Myalgia	140 (63.6%)	40 (68.9%)	100 (61.7%)	0.33
Arthralgia	68 (30.9%)	21 (36.2%)	47 (29.0%)	0.31
Neurogenic pain	39 (17.7%)	12 (20.6%)	27 (16.6%)	0.49
Gastrointestinal disorders	79 (35.9%)	16 (27.5%)	63 (38.8%)	0.12

Categorical variables are presented as counts and percentages. Quantitative variables are presented as medians and interquartile ranges. PCS: post-COVID syndrome; PEM: post-exertional malaise; PCS/PEM+: group of patients with PEM; PCS/PEM−: group of patients without PEM; FSS: Fatigue Severity Scale; POTS: postural orthostatic tachycardia syndrome; MCAS: mast cell activation syndrome; MCAD: mast cell activation disorder.

**Table 2:** Multivariate analysis of variables correlated with the occurrence of post-exertional malaise in patients with post-COVID syndrome.

	*p*-Value	Odds Ratio
Gender	0.51	0.75 (95% CI: 0.32–1.74)
Age at COVID-19 onset	0.30	1.02 (95% CI: 0.99–1.05)
Time from COVID-19 onset to post-COVID-19 syndrome diagnosis	0.25	1.55 (95% CI: 0.74–3.25)
COVID onset in 2020/2021	0.006	5.68 (95% CI: 1.66–19.45)
Vaccinated patients	0.06	2.68 (95% CI: 0.95–7.58)
POTS	0.47	1.63 (95% CI: 0.43–6.30)
OH	0.06	2.68 (95% CI: 0.95–7.58)
Palpitations	0.11	1.89 (95% CI: 0.86–4.12)
FSS score	0.007	2.07 (95% CI: 1.22–3.50)

Multivariate analysis was conducted using binary logistic regression. The dependent variable was the occurrence of post-exertional malaise. The included variables were gender, age at COVID-19 onset (as continuous data), time from COVID-19 onset to post-COVID-19 syndrome diagnosis (as a dichotomous variable above or below the median of 15 months), and variables significantly associated with PEM in univariate analysis: number of vaccinated patients, POTS (postural orthostatic tachycardia syndrome), OH (orthostatic hypotension), palpitations, and FSS (Fatigue Severity Scale) score. Odds ratios and 95% confidence intervals (CIs) are reported only for variables that reached statistical significance in the final multivariate logistic regression model.

## Data Availability

The data presented in this study are available on request from the corresponding author. The data are not publicly available due to privacy restrictions.
